# Serum Extracellular Vesicle-Derived circHIPK3 and circSMARCA5 Are Two Novel Diagnostic Biomarkers for Glioblastoma Multiforme

**DOI:** 10.3390/ph14070618

**Published:** 2021-06-27

**Authors:** Michele Stella, Luca Falzone, Angela Caponnetto, Giuseppe Gattuso, Cristina Barbagallo, Rosalia Battaglia, Federica Mirabella, Giuseppe Broggi, Roberto Altieri, Francesco Certo, Rosario Caltabiano, Giuseppe Maria Vincenzo Barbagallo, Paolo Musumeci, Marco Ragusa, Cinzia Di Pietro, Massimo Libra, Michele Purrello, Davide Barbagallo

**Affiliations:** 1 Department of Biomedical and Biotechnological Sciences, Section of Biology and Genetics Giovanni Sichel, University of Catania, 95123 Catania, Italy; michelestella7@gmail.com (M.S.); caponnettoangela@gmail.com (A.C.); barbagallocristina@unict.it (C.B.); rosaliabattaglia04@gmail.com (R.B.); mirabella.federica.91@gmail.com (F.M.); mragusa@unict.it (M.R.); dipietro@unict.it (C.D.P.); purrello@unict.it (M.P.); 2 Department of Biomedical and Biotechnological Sciences, Section of Pathology, University of Catania, 95123 Catania, Italy; luca.falzone@unict.it (L.F.); peppeg9305@gmail.com (G.G.); mlibra@unict.it (M.L.); 3 Department of Medical, Surgical Sciences and Advanced Technologies “G.F. Ingrassia”, Section of Anatomic Pathology, University of Catania, 95123 Catania, Italy; giuseppe.broggi@gmail.com (G.B.); rosario.caltabiano@unict.it (R.C.); 4 Department of Medical, Surgical Sciences and Advanced Technologies “G.F. Ingrassia”, Neurological Surgery, Policlinico “Rodolico-San Marco” University Hospital, University of Catania, 95123 Catania, Italy; roberto.altieri.87@gmail.com (R.A.); cicciocerto@yahoo.it (F.C.); gbarbagallo@unict.it (G.M.V.B.); 5 Interdisciplinary Research Centre on the Diagnosis and Therapy of Brain Tumors, University of Catania, 95123 Catania, Italy; 6 Department of Physics and Astronomy, University of Catania, 95123 Catania, Italy; paolo.musumeci@ct.infn.it; 7 Research Center for Prevention, Diagnosis and Treatment of Cancer, University of Catania, 95123 Catania, Italy

**Keywords:** glioblastoma multiforme, circRNAs, extracellular vesicles, diagnostic biomarkers

## Abstract

Glioblastoma multiforme (GBM) is the most frequent and deadly human brain cancer. Early diagnosis through non-invasive biomarkers may render GBM more easily treatable, improving the prognosis of this currently incurable disease. We suggest the use of serum extracellular vesicle (sEV)-derived circular RNAs (circRNAs) as highly stable minimally invasive diagnostic biomarkers for GBM diagnosis. EVs were isolated by size exclusion chromatography from sera of 23 GBM and 5 grade 3 glioma (GIII) patients, and 10 unaffected controls (UC). The expression of two candidate circRNAs (circSMARCA5 and circHIPK3) was assayed by droplet digital PCR. CircSMARCA5 and circHIPK3 were significantly less abundant in sEVs from GBM patients with respect to UC (fold-change (FC) of −2.15 and −1.92, respectively) and GIII (FC of −1.75 and −1.4, respectively). Receiver operating characteristic curve (ROC) analysis, based on the expression of sEV-derived circSMARCA5 and circHIPK3, allowed us to distinguish GBM from UC (area under the curve (AUC) 0.823 (0.667–0.979) and 0.855 (0.704 to 1.000), with a 95% confidence interval (CI), respectively). Multivariable ROC analysis, performed by combining the expression of sEV-derived circSMARCA5 and circHIPK3 with preoperative neutrophil to lymphocyte (NLR), platelet to lymphocyte (PLR) and lymphocyte to monocyte (LMR) ratios, three known diagnostic and prognostic GBM markers, allowed an improvement in the GBM diagnostic accuracy (AUC 0.901 (0.7912 to 1.000), 95% CI). Our data suggest sEV-derived circSMARCA5 and circHIPK3 as good diagnostic biomarkers for GBM, especially when associated with preoperative NLR, PLR and LMR.

## 1. Introduction

Glioblastoma multiforme (GBM) is the most common malignant and highly aggressive primary brain tumor in adults, with a median post-diagnosis overall survival of 16 months, despite the current therapeutical approach based on surgical resection, followed by chemotherapy and radiotherapy [1,2,3]. According to the World Health Organization (WHO) classification, GBM is a grade IV glioma, with several unique features that characterize its aggressiveness and unresponsiveness to therapy [1,4]. GBM is typically a heterogeneous cancer, characterized by a wide range of genetic and epigenetic intra- and inter-tumor variability [5,6]; its microenvironment is made up of several cell types: glioma cells, glioma stem cells (GSC), stromal cells that include resident glial cells such as oligodendrocytes, astrocytes, ependymal cells, and microglia and infiltrating immune cells such as monocytes, macrophages, and lymphocytes [7]. Glioma cells reside in a niche of stromal cells and communicate with them to establish a cancer progression-promoting environment through cell–cell gap junctions, the secretion of effector biomolecules including growth factors, cytokines, chemokines, and extracellular vesicles (EVs) [2,8,9,10].

EVs are a heterogeneous group of lipid bilayer-delimited particles that can be classified into various subtypes such as exosomes, ectosomes, microvesicles, and apoptotic bodies, based on their biogenesis (budding or active secretory pathway), size (ranging from 30 to 2000 nm), and molecular structure [11,12,13]. EVs are involved in several biological functions thanks to their role in the transfer of different species of RNAs and other bioactive molecules from donor to proximal or distant recipient cells through body fluids [14,15,16,17,18,19,20]. The number and cargo of EVs may change according to physiological or pathological conditions; in several cancers, it has been shown that the quality and quantity of their cargo appear to be altered and differentially expressed (DE) molecules (mainly, RNAs and proteins) have been suggested as diagnostic or prognostic biomarkers of disease [21,22,23,24,25].

Circular RNAs (circRNAs) are covalently closed RNA molecules, mostly non-coding, mainly synthesized starting from pre-mRNA produced by protein-coding genes through back-splicing [26,27]. Their particular structure renders them intrinsically resistant to degradation mediated by exonucleases and, consequently, more stable than their linear isoform counterparts, both inside and outside cells [28]. Most studies describe circRNAs as involved in the regulation of gene expression (e.g., acting as microRNA (miRNA) or RNA binding protein (RBP) sponges) [29,30,31,32]. CircRNAs show species, tissue, and developmental stage-specific expression patterns; in addition to being expressed inside cells (especially in the cytoplasm), they have been found as cargo components of EVs [33,34,35,36]. The expression of circRNAs has been found to be dysregulated in several pathological conditions, including gliomas and GBM [34,37,38,39,40,41,42,43]. We recently characterized circSMARCA5 as a tumor-suppressive circRNA, downregulated in GBM tissue when compared to normal brain parenchyma; its expression is inversely correlated with: (i) overall and progression-free survival of GBM patients; (ii) pro- to anti-angiogenic VEGFA mRNA isoform ratio; and (iii) microvascular density of GBM tissue [31]. Mechanistically, we determined that circSMARCA5 physically interacts with the oncoprotein SRSF1 through its GAUGAA RNA motif: mutation of this motif determined a significant decrease in the binding between the two molecules with related downstream effects on GBM cell migration and angiogenic potential [32,44]. To further characterize the molecular phenotype of GBM patients, we assayed the expression of circSMARCA5 and other circRNAs known to be dysregulated in GBM in EVs isolated from serum (sEVs) of GBM patients and compared these data to those from unaffected controls (UC).

## 2. Results

### 2.1. Characterization of EVs Isolated from GBM and Control Serum

**Figure 1:**
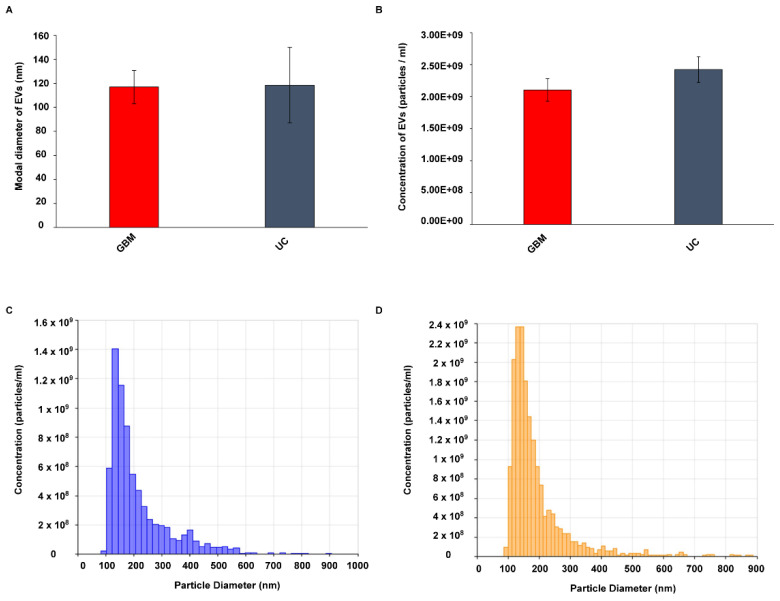
Bar graphs showing (**A**) modal diameter (nm) and (**B**) concentration (particles/mL) of sEVs isolated from GBM and UC. Data are represented as mean ± SEM of the measurements performed by tunable resistive pulse sensing (TRPS) on three randomly chosen GBM and control samples. Plots showing concentration (particles/mL, y-axis) and size (nm) distribution (x-axis) of sEVs isolated from a representative UC and GBM sample are shown in (**C**,**D**), respectively.

qNano Gold analysis performed on sEVs isolated from GBM and UC samples revealed: (i) a mean modal diameter of 117 and 118.5 nm (Figure 1A); (ii) a mean concentration of 2.10 × 10^9^ and 2.42 × 10^9^ particles/mL (Figure 1B), respectively; and (iii) a majority of particles sized between 100 and 200 nm (Figure 1C,D). Qualitative analysis of sEVs, performed through immunogold labeling followed by transmission electron microscopy (TEM), showed their positivity to the tetraspanin CD81 (Supplementary Figure S1).

### 2.2. circSMARCA5 and circHIPK3 Are Downexpressed in sEVs from GBM with Respect to Grade 3 Glioma (GIII) and UC

**Figure 2:**
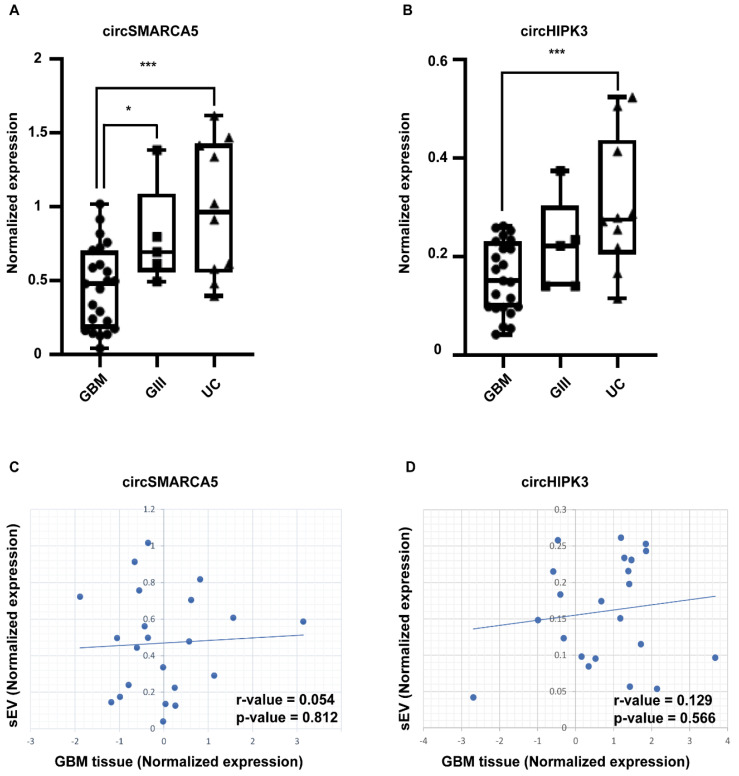
CircSMARCA5 (**A**) and circHIPK3 (**B**) expression in sEVs isolated from GBM, GIII and UC. Data are represented as box and whisker plots of normalized ddPCR data. * *p*-value < 0.05; *** *p*-value < 0.001, Student’s *t*-test (n_GBM_ = 23; n_GIII_ = 5; n_UC_ = 10). Correlation between the expression of sEV-derived and GBM tissue-derived circSMARCA5 (**C**) and circHIPK3 (**D**) is reported as a dot plot. R-values were calculated through Pearson’s correlation test; *p*-values were calculated according to r-values and the number of clinical specimens analyzed.

Among the circRNAs known to be DE in GBM, circSMARCA5 and circHIPK3 appeared as the top two most expressed in sEV cargo, in physiological conditions, according to exoRBase (Table 1). Droplet digital PCR (ddPCR) confirmed the expression of both circRNAs in sEVs isolated from UC and revealed their significant underexpression in sEVs from GBM (circSMARCA5 and circHIPK3 fold-change (FC) = −2.15 and −1.92, *p*-value = 0.00028 and 0.00034, Student’s *t*-test, respectively) (Figure 2A,B). sEV-derived circSMARCA5 and circHIPK3 were also underexpressed in GBM as compared to GIII (FC = −1.75 and −1.4, *p*-value = 0.026 and 0.10, Student’s *t*-test, respectively) (Figure 2A,B). The expression of circSMARCA5 and circHIPK3 within EVs from GBM sera did not significantly correlate with their expression assayed in matched GBM tissues, derived from the same patients (Figure 2C,D).

### 2.3. sEV-Derived circSMARCA5 and circHIPK3 Expression Correlates with the Level of Preoperative Hematological Diagnostic and Prognostic Inflammatory Markers of GBM

**Figure 3:**
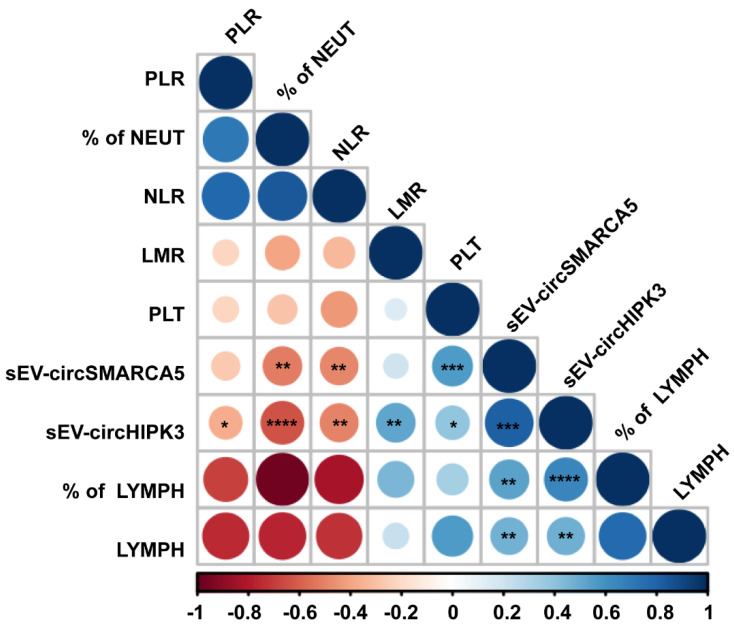
Correlogram showing correlations between the expression of sEV-derived circSMARCA5 and circHIPK3 and hematological data. Scale-bar shows r-values calculated through Pearson’s correlation test. The size of each circle is directly proportional to the statistical significance that is shown for some correlation of interest through one (* *p*-value < 0.05), two (** *p*-value < 0.01), three (*** *p*-value < 0.001) or four (**** *p*-value < 0.0001) asterisks. (NEUT = Neutrophils, PLT = Platelet count, LYMPH = Lymphocytes).

Hematological analysis revealed a significant increase in the percentage of neutrophils (among white blood cells) and of two known diagnostic and prognostic inflammatory markers of GBM (the neutrophil to lymphocyte ratio (NLR) and the platelet to lymphocyte ratio (PLR)—see Discussion) in GBM patients as compared to UC (Supplementary Figure S2). The percentage (among white blood cells) and the count lymphocytes, as well as the lymphocyte to monocyte ratio (LMR) (another marker whose low level is linked to a dismal prognosis of GBM patients—see Discussion), significantly decreased in GBM patients as compared to UC (Supplementary Figure S2). The amount of sEV-derived circSMARCA5 and circHIPK3 positively correlated with the number of platelets, the percentage and the total number of lymphocytes, while it negatively correlated with the percentage of neutrophils and NLR; moreover, PLR and LMR negatively and positively correlated with the amount of sEV-derived circHIPK3, respectively (Figure 3).

### 2.4. Serum EV-Derived circSMARCA5 and circHIPK3 Can Be Considered Reliable GBM Diagnostic Biomarkers

**Figure 4:**
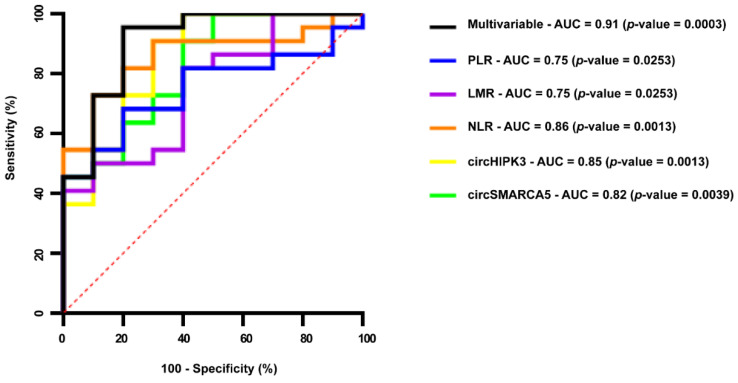
Univariable and multivariable receiver operating characteristic (ROC) curve analysis based on the expression of sEV-derived circSMARCA5 and circHIPK3, NLR, PLR, LMR and their combination.

Receiver operating characteristic curve (ROC) analysis based on the expression of sEV-derived circSMARCA5 and circHIPK3 allowed us to distinguish GBM from UC (area under the curve (AUC) 0.823 (0.667–0.979) and 0.855 (0.704 to 1.000), with a 95% confidence interval (CI), respectively). Multivariable ROC analysis, performed by combining the expression of sEV-derived circSMARCA5 and circHIPK3 with preoperative NLR, PLR and LMR, allowed an improvement in the GBM diagnostic accuracy (AUC 0.901 (0.7912 to 1.000), 95% CI) (Figure 4).

## 3. Discussion

The interest in finding new non-invasive cancer biomarkers for diagnostic, prognostic and response-to-therapy purposes has been increasing in the last few years [45,46,47,48]. This is one of the first studies that focuses on circRNAs as non-invasive diagnostic biomarkers, detectable in GBM patients’ liquid biopsies: to the best of our knowledge, only Chen et al. published a paper on three plasmatic circRNAs (circFOXO3, circ_0029426, and circ-SHPRH) with a diagnostic value for GBM [49]. Due to their intrinsic resistance to exonucleases and resulting high stability, circRNAs, and especially EV-associated circRNAs, may be considered very good candidate biomarkers [42,45]. Here, we focused on circSMARCA5 and circHIPK3: the first is a known tumor suppressor, whereas the second is an oncogenic circRNA in GBM cells [30,31,32,50]. In addition, both circRNAs have been detected in sEVs, according to ExoRBase, a database storing circRNA expression data in sEVs in physiological and pathological conditions [51]. CircSMARCA5 has been defined as a biomarker for atherosclerosis, hepatocellular carcinoma and gastric cancer, and has been found in plasma, serum and whole blood [36,52,53,54,55,56]. CircHIPK3 has been detected in human umbilical cord mesenchymal stem cell-derived exosomes, and its release to ischemic muscle seems to contribute to the repair of damaged tissue [57]. CircHIPK3 has also been suggested as a diagnostic and prognostic biomarker, detectable in the serum exosomes and whole serum of colorectal cancer, chronic myeloid leukemia and nasopharyngeal carcinoma patients [58,59,60]. Both circRNAs were significantly less abundant in sEVs from GBM when compared to UC, and their expression appeared to be significantly positively correlated. Our data also suggest that the differential expression of sEV-derived circSMARCA5 and circHIPK3 is independent from that observed in GBM tissue. The lack of this correlation may be due to dysregulated EV production and EV cargo from non-tumoral cells such as brain or blood cells in GBM patients [61]. The latter hypothesis is supported by the positive correlation between the expression of both circRNAs and the number of platelets, as well as the percentage and the total number of lymphocytes that we found in our cohort of GBM patients and UC. It is known that platelets and lymphocytes are among the major contributors of EVs in serum [62,63], and both cell types express circSMARCA5 and circHIPK3 [64,65,66]. Furthermore, GBM cells are able to “educate” platelets and other blood formed elements, modifying their transcriptome and splicing pattern [67,68], which suggests that blood cells, and especially platelets, lymphocytes or both, may contribute to the dysregulated expression of sEV-derived circSMARCA5 and circHIPK3 that we observed in GBM patients with respect to UC. The diagnostic and clinical relevance of the altered expression of sEV-derived circSMARCA5 and circHIPK3 in GBM is supported by their correlation with NLR, PLR and LMR. The diagnostic and prognostic value of these inflammatory markers in GBM has been largely described in the literature [69,70,71,72]. According to these data, a multivariable ROC analysis, performed by combining the expression of sEV-derived circSMARCA5 and circHIPK3 with NLR, PLR and LMR, allowed an improvement in the diagnostic value as compared to a univariable analysis: this is in accordance with other studies, confirming the strength of a diagnosis based on a multi-biomarker panel [73,74,75].

## 4. Materials and Methods

### 4.1. Biological Samples

Sera from 23 GBM and 5 GIII patients and 10 UC were obtained from whole blood collected in sterile BD Vacutainer^®^ SST II Advance tubes with clot activator (Becton Dickinson and Company, NJ, USA). Blood samples were collected both from GBM patients, before neurosurgery, and from controls, during check-ups at the day hospital at the Azienda Ospedaliero-Universitaria “Policlinico Vittorio Emanuele”, Catania, Italy. Once the whole blood samples clotted, serum was collected and centrifuged twice at 2150× *g* for 15′ at room temperature to pellet potential residual blood cells. Supernatants were stored at −80 °C until further processing. GBM tissue biopsies were collected from the same patients and stored at −80 °C until further processing, as previously described [31]. All patients were diagnosed for GBM by at least three experienced pathologists, according to the 2016 WHO criteria [4]. Informed consent was obtained from all subjects involved in the study. The study was approved by the local ethics committee of the Azienda Ospedaliero-Universitaria “Policlinico-Vittorio Emanuele”, Catania, Italy. Age, sex and clinical features of the GBM patients and UC are reported in Table 2.

### 4.2. EVs Isolation and Quantification

Hemolysis of serum samples was evaluated through spectrophotometric analysis. The absorbance of a representative 100 µL aliquot from each sample was measured at two wavelengths (λ = 541 nm and 576 nm) using the Synergy 2-BioTek plate reader (Agilent Technologies, Inc., Santa Clara, CA, USA): only samples with an absorbance value <0.2 were considered non-hemolyzed and were used for further processing [76]. Briefly, a 1 mL aliquot from non-hemolyzed sera was thawed at 4 °C and centrifuged at 1500× *g*, 10′, 4 °C; the supernatant was centrifuged again at 8000× *g*, 10′, 4 °C to remove potential debris: supernatants from the second centrifugation were used for sEV isolation. sEVs were isolated from 500 uL of serum by using SEC, through qEV original/70nm chromatography columns (IZON Science, Christchurch, New Zealand). Sterile PBS, filtered through 0.2 µm filters, was used as eluent and pooled fractions 7–10 were collected in a final volume of 2 mL and stored at −80 °C until further processing, according to the manufacturer’s instructions. For three randomly chosen GBM and control samples, an aliquot of 100 µL was assayed by TRPS through qNano Gold (IZON Science) to determine the size and the concentration of isolated EVs. TRPS data analysis was performed through qNANO Control Suite Software v. 3.4 (IZON Science).

### 4.3. RNA Extraction, cDNA Synthesis and ddPCR

EVs were lysed with five volumes of QIAzol Lysis Reagent (Qiagen, Venlo, Netherlands, EU) and RNA was extracted using miRNeasy Mini Kit (Qiagen), following the manufacturer’s instructions. cDNA was obtained using SuperScript™ II Reverse Transcriptase (ThermoFisher Scientific, Waltham, MA, USA), according to the manufacturer’s protocol. cDNA was amplified through ddPCR using QX200 ddPCR EvaGreen Supermix™ (Bio-Rad Laboratories, Inc., Hercules, CA, USA), as previously described [77,78]. ddPCR data analysis was performed using QuantaSoft™ Analisys Pro Software V1.0. GAPDH was used as endogenous control RNA. GBM tissues were processed as previously described to obtain RNA [31]. Purified RNA was amplified through qRT-PCR using Power SYBR^®^ Green RNA-to-CT™ 1-Step Kit (ThermoFisher Scientific), in a 7900HT Fast real-time PCR system (ThermoFisher Scientific). Primer sequences used in this study are reported in Table 3.

### 4.4. Statistical Analysis

Differential expression between GBM and control groups, ROC curves and AUCs were calculated by using IBM^®^ SPSS^®^ v. 23 and GraphPad Prism v. 8.0.2. In particular, binary logistic regression was used to build a probabilistic model by combining the expression of multiple markers: ROC curve was generated, accordingly. Normal distribution of gene expression data was assessed by Kolmogorov–Smirnov test of normality. Differential expression analysis between GBM and control groups was defined through Student’s two-tailed *t*-test. Pearson’s correlation coefficient was calculated to define positive and negative correlations. *p*-values < 0.05 were considered statistically significant.

## 5. Conclusions

Our data convincingly suggest sEV-derived circSMARCA5 and circHIPK3 as new good diagnostic GBM biomarkers, especially when combined with preoperative NLR, PLR and LMR data.

## Figures and Tables

**Table 1:** Candidate DE circRNAs in gliomas and their expression in sEVs from healthy individuals according to exoRBase (http://www.exorbase.org/exoRBase/toIndex, access on April 2020). Median expression data are reported as reads per million (RPM).

circBase ID	exoRBase ID	Gene Symbol	Median Expression in Serum EVs from Healthy Individuals (RPM)	Differential Expression (Glioma Tissue vs. Normal Brain Parenchyma)
hsa_circ_0001445	exo_circ_000006	SMARCA5	16,237.253	Downregulated (PMIDs: 29415469; 30736462)
hsa_circ_0000284	exo_circ_000027	HIPK3	3193.315	Upregulated (PMID: 30057315)
hsa_circ_0001009	exo_circ_000142	FANCL	1448.15	Downregulated (PMID: 25921068)
hsa_circ_0001730	exo_circ_000064	EPHB4	503.7355	Upregulated (PMID: 26873924)
hsa_circ_0003496	exo_circ_000134	UBAP2	375.249	Upregulated (PMID: 29920451)
hsa_circ_0122319; hsa_circ_0067682	exo_circ_000449	PLOD2	339.669	Upregulated (PMID: 26873924)
hsa_circ_0008386	exo_circ_000476	LRRFIP2	309.193	Downregulated (PMID: 29920451)
hsa_circ_0000915	exo_circ_000422	FKBP8	225.519	Downregulated (PMID: 26873924)
hsa_circ_0074371	exo_circ_000452	ARHGAP26	174.8015	Upregulated (PMID: 30388035)
hsa_circ_0001819	exo_circ_001051	UBR5	159.979	Upregulated (PMID: 29920451)
hsa_circ_0000199	exo_circ_000834	AKT3	105.7865	Downregulated (PMID: 26873924)

**Table 2:** Demographic data of the case and control cohorts involved in the study.

Type of Samples	N° of Samples	Mean Age (Years) ± StdDev	Sex	
			M	F
GBM	23	65.2 ± 11.1	13	10
GIII	5	46.8 ± 15.4	1	4
UC	10	61.8 ± 10.20	5	5

**Table 3:** Primer sequences used in this study.

Transcript Name	Primer Sequence
*GAPDH*	Fw: 5′-GTCAGCCGCATCTTCTTTTG-3′
	Rev: 5′-GCGCCCAATACGACCAAATC-3′
*TBP*	Fw: 5′-ACTTGACCTAAAGACCATTGCA-3′
	Rev: 5′-GGCTCTCTTATCCTCATGATTACC-3′
circSMARCA5	Fw: 5′-ACAATGGATACAGAGTCAAGTGTT-3′
	Rev: 5′-CACATGTGTTGCTCCATGTCT-3′
circHIPK3	Fw: 5′-GGTCGGCCAGTCATGTATCA-3′
	Rev: 5′-AGGCCATACCTGTAGTACCGA-3′

## Data Availability

Data is contained within the article and supplementary material.

## Supplementary Materials

The following are available online at https://www.mdpi.com/article/10.3390/ph14070618/s1, Figure S1: Transmission electron microscopy (TEM) micrograph of CD81-immunolabelled EVs, Figure S2: Amount of formed elements of blood in GBM and UC.
