# Supplementary material for: Serum Extracellular Vesicle-Derived circHIPK3 and circSMARCA5 Are Two Novel Diagnostic Biomarkers for Glioblastoma Multiforme
Source: Pharmaceuticals (Basel). 2021 Jun 27;14(7):618. doi: 10.3390/ph14070618 (PMC8308516; doi:10.3390/ph14070618)
Supplement: Supplementary file 1 [file pharmaceuticals-14-00618-s001.zip › pharmaceuticals-1240824-SI.pdf]

Supplementary figures

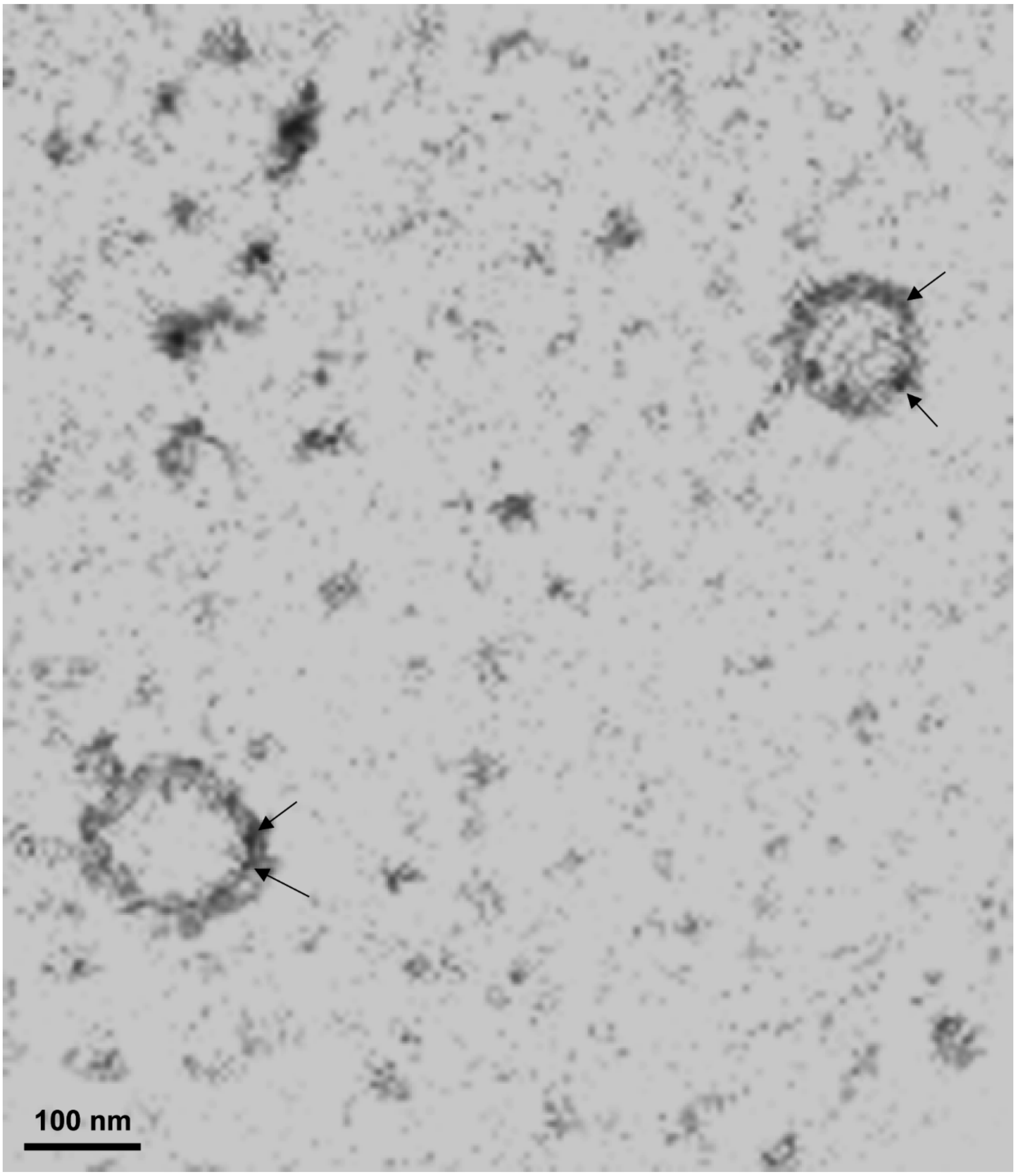

Supplementary Figure S1. Transmission electron microscopy (TEM) micrograph, showing positivity of isolated sEVs to the tetraspanin CD81. CD81 expression on the sEV’s membrane is highlighted by arrows.

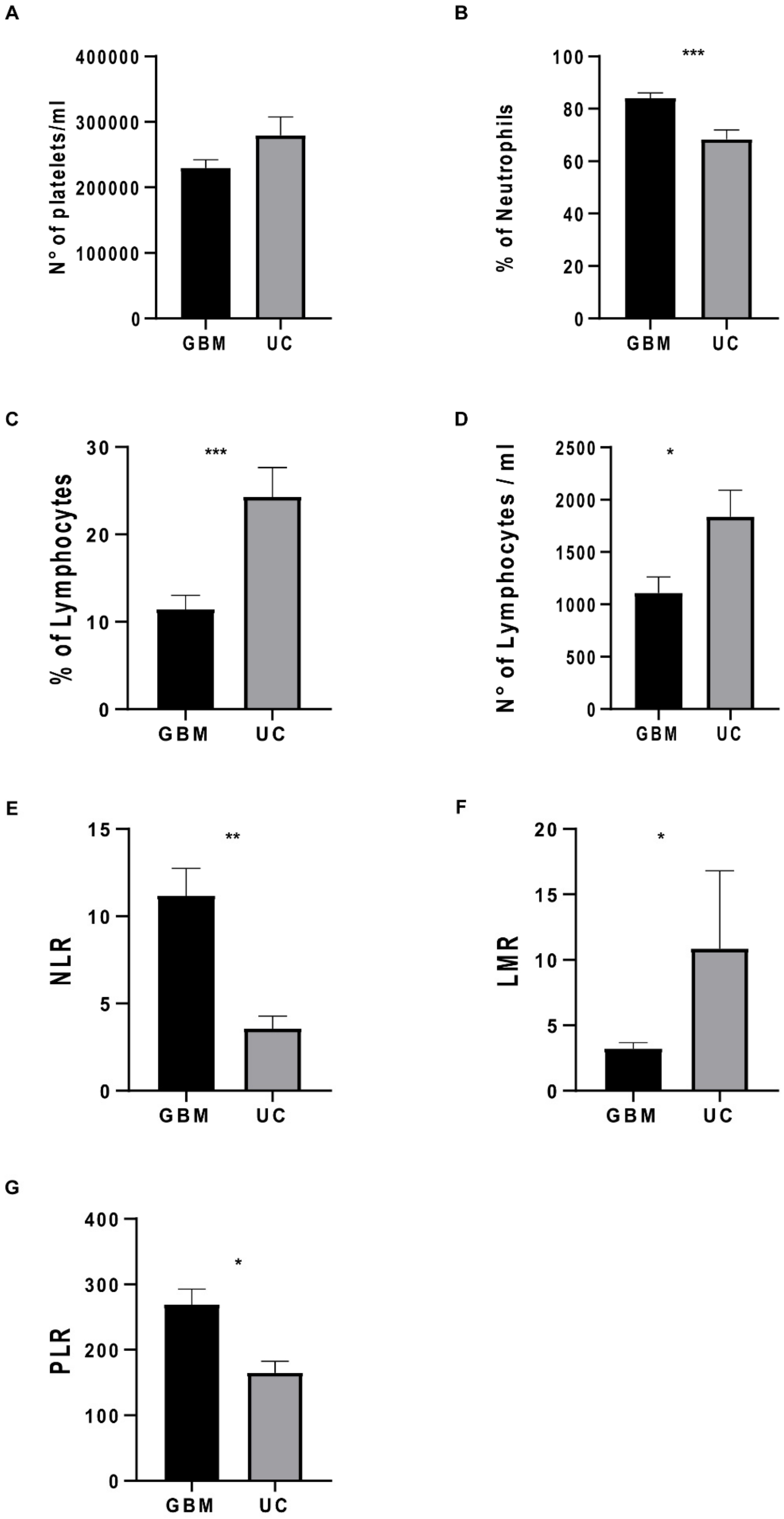

Supplementary Figure S2. Bar-graphs showing the platelet count (A), the percentage of neutrophils (B) and lymphocytes (C) (among white blood cells), lymphocyte count (D), preoperative neutrophil to lymphocyte (NLR) (E), lymphocyte to monocyte (LMR) (F) and platelet to lymphocyte (PLR) (G) ratios in GBM and UC. *** p-value < 0.001; ** p-value < 0.01; * p-value < 0.05, Student's t-test (n_{GBM} = 23; n_{UC} = 10).

Supplementary materials and methods

CD81 immunolabelling and transmission electron microscopy (TEM)

CD81 immunolabelling and TEM were performed as previously described [PMID: 30643155]. Briefly, 30 µl of one representative sample of isolated sEVs were fixed in 50 µl of 3% formaldehyde–0.1% glutaraldehyde. Adsorption of sEVs occurred on formvar copper-coated nickel grids (Electron Microscopy Sciences, Fort Washington, PA) by drying them for 20 ′. The grid, washed in PBS, was negatively stained with 4% uranyl acetate for 5 ′. sEVs were adsorbed on the down side of the grid. The grid was rinsed 2 times for 2 ′ with PBS and transferred in a TBS (Tris buffered saline pH 7.4) solution containing 1% BSA (bovine serum albumin) (TBS/BSA) for 10 ′ at room temperature, for immunoelectron microscopy labelling. Then the grid was incubated in 5% BSA (blocking solution) for 90 ′ at room temperature, rinsed with PBS and incubated in a humid chamber overnight at 4 °C with a mouse monoclonal antibody CD81 (Santa Cruz Biotechnology, Heidelberg, Germany) in a dilution 1:20 with TBS/BSA. Three washes (3 ′ each) with TBS/ BSA were carried out, and after that, the grid was stained with a 10 nm gold-labelled secondary antibody antimouse IgG (Sigma- Aldrich, S.r.l., Milan, Italy) in a dilution 1:5 with TBS/BSA at 37 °C for 1 h in the dark. The grid was rinsed 2 times for 2 ′ with TBS/ BSA, 2 times for 2 ′ with H_2O and fixed with 1.5% glutaraldehyde in PBS for 10 ′ at room temperature. After one more rinse with H_2O, the grid was stained with 4% uranyl acetate for 5 ′, then it was incubated with 0.13% methylcellulose and 0.4% uranyl acetate on ice for 10 ′ minutes and allowed to air-drying. sEVs were observed through JEOL JEM2010 TEM operating at 200 kV.
